# Epidemiology of Severe Cutaneous Adverse Drug Reaction and Its HLA Association among Pediatrics

**Published:** 2019

**Authors:** Hossein Esmaeilzadeh, Shirin Farjadian, Soheila Alyasin, Hamid Nemati, Hesamodin Nabavizadeh, Elmira Esmaeilzadeh

**Affiliations:** a *Allergy Research Center, Shiraz University of Medical Sciences, Shiraz, Iran. *; b *Department of Allergy and Clinical Immunology, Shiraz University of Medical Sciences, Shiraz, Iran.*; c *Department of Immunology, Shiraz University of Medical Sciences, Shiraz, Iran. *; d *Shiraz Neuroscience Research Center, Shiraz University of Medical Sciences, Shiraz, Iran. *; e *Department of Internal Medicine, Division of Rheumatology, Shiraz University of Medical Sciences, Shiraz, Iran.*

**Keywords:** DHR, SCAR, STS/TEN, DRESS, AGEP, HLA

## Abstract

Severe cutaneous adverse drug reaction (SCAR) is considered to be a multifactorial drug side effect. This study was designed to investigate the epidemiology and human leukocyte antigen (HLA)-A and -B gene polymorphisms in pediatric patients with SCAR admitted in tertiary referral center, southwestern of Iran from 2013 to 2017. Demographic data, past allergy and autoimmune history, clinical presentations, drugs confirmed to be the cause of SCAR as well as its therapy were reviewed for each patient. HLA-A and -B allele frequencies were determined in 40 of the patients using polymerase chain reaction based on sequence specific primers (PCR-SSP) and compared with 40 healthy individuals as control group. Sixty-one patients with mean age of 6 years old and boy to girl ratio was 1.2/1 in this study. The most common type of SCAR in our patients was Steven Johnson Syndrome (SJS)/Toxic Epidermal Necrosis (TEN) mainly caused by beta-lactam antibiotics. Carbamazepine was the second cause of drug–induced SCAR. Moreover, HLA-A*02:01 and A*51:01 were related to the increased risk of SCAR while A*11:01 seemed to be protective against SCAR. HLA-A*02:01, HLA-A*24:02, and HLA-B*51:01 showed associations to the increased risk of SJS. Based on our results, beta-lactam antibiotics and antiepileptic drugs are the most common causes of severe adverse drug reaction in southwestern Iranian pediatric patients. Moreover, some HLA-A alleles can influence risk of SCAR.

## Introduction

Drug hypersensitivity reactions (DHRs) refer to unpredictable adverse effect of drugs in therapeutic doses tolerated by healthy indivituals (1, 2). DHR is an immunologic reaction classified to IgE–mediated (Immediate) and non-IgE–mediated (delayed) subtypes (3). Although DHR is estimated to include one third of adverse drug reactions (ADRs), there are just limited epidemiologic data in pediatrics (1, 4). 

The most common organ involved in DHR is skin characterized by a range of symptoms varied from benign skin eruption to severe presentations known as severe cutaneous adverse drug reactions (SCARs) such as acute generalized exanthematous pustulosis (AGEP), drug reaction with eosinophilia and systemic symptoms (DRESS), Stevens-Johnson syndrome (SJS) and toxic epidermal necrolysis (TEN) (5, 6). The most common drugs causing SCARs were detected to be antiepileptic drugs, antibiotics, allopurinol, and nonsteroidal anti-inflammatory drugs (NSAIDs) (7, 8). 

Although the exact pathophysiology of SCARs has not been established yet, most of recent studies suggested a key role of T cell–mediated delayed type immune reactions with possible interaction between human leukocyte antigen (HLA) molecules and drug metabolites (9). In this regard, some studies suggested a role for variants of HLA and other genes such as CYP2C, ABCB1, and CYP3A4 in explaining the variability and unpredictability of DHR presentations among different individuals (10, 11). This study was designed to investigate the most common drugs leading to SCAR in southwestern Iranian pediatrics. The relation between HLA-A and -B alleles and SCAR was also determined.

## Experimental

*Patients Selection*

Sixty-one patients were recruited to this study during a period of 2013 to 2017 from those patients admitted by diagnosis of SCAR at Namazi Hospital of Shiraz University of Medical Sciences, Shiraz, southwestern Iran. Sever cutaneous reactions due to sepsis, vasculitis, or not drug induced lesions were excluded. Demographic data with past medical history of the patients, list of drugs taken by the patients before admission, clinical presentations, laboratory findings and DHR treatment were recorded for each patient. Genotyping of HLA-A and -B was done for 40 of the patients and 40 healthy controls from the same geographic region.

SCAR was diagnosed and classified to four major types: DRESS, SJS, TEN, and AGEP. The diagnosis of DRESS was made using Registry of Severe Cutaneous Reactions (RegiSCAR) scoring system (12). In this scoring system, the diagnosis is based on the presence of mucocutaneous skin rash, fever, lymphadenopathy, and hematologic disorders such as eosinophilia and scores more than five are diagnosed as DRESS. The diagnosis was made clinically. At least two acute mucosal involvements, skin rash such as maculae, target like lesions, bullae, positive Nikolsky sign as well as epidermal detachment less than 10% of body surface area was considered as SJS. Similar clinical manifestation with body surface area involvement more than 30% was considered as TEN (11). The presence of non-follicular, pustular lesions less than 5 mm on an erythematous skin appearing few days after first dose of drug was considered as clinical diagnosis for AGEP. Based on EuroSCAR scoring system, patients with score 8-12 were diagnosed as definite AGEP (3). 

*HLA-A and -B genotyping*

Genomic DNA was extracted from 200 µL of each blood sample using a commercial kit (Genet Bio, Nonsan, South Korea). HLA-A and -B genotyping was performed by polymerase chain reaction using sequence specific primers (PCR-SSP) method using a commercial kit (Texas BioGene, Texas, USA) based on manufacturer’s suggested method. Then, PCR products were electrophoresed on 2% agarose gel containing gel red and visualized under UV light; afterwards, the pattern of specific bands was analyzed by a related software included in the kit.

*Statistical Analysis*

HLA Allele frequencies were compared between patients and controls with the chi-squared test or Fisher’s exact test, and odds ratio (OR) with a 95% confidence interval (CI) was calculated using Epi Info v.7 and *P* < 0.05 was considered significant. 

## Results

To investigate the main drugs, lead to SCARs in southwestern Iranian pediatrics, 61 patients (34 boys and 27 girls) with mean age of 6 ±5 years and age range 2 months to 17 years. The patient characteristics are summarized in Table 1. The frequency of drugs led to SJS, TEN, DRESS or AGEP presentations in our patients is shown in Table 2. As shown, antiepileptic drugs (36%) and antibiotics (44.3%) were the main cause of SCARs.

The frequency of HLA-A and -B alleles in patients with SCARs**, **among patients with hypersensitivity to anticonvulsants, beta-lactam and non-beta lactam antibiotics, as well as in patients with SJS compared to healthy controls is presented in Tables 3, 4 and 5, respectively.

## Discussion

Severe cutaneous adverse reactions (SCAR) are delayed type hypersensitivities presented with severe clinical manifestations such as SJS, TEN, DRESS, or AGEP (13). There are just limited data about the epidemiology, etiology, clinical presentations, and outcome of SCAR. 

The results of this study on 61 pediatric patients with SCARs revealed that SJS with a frequency of 81.7% was the most common subtype. In contrast to the reports from Turkey, Malaysia, and Australia which identified DRESS as the most common SCAR subtype (8, 14 and 15), our data were in the line with the results of a meta-analysis from China (16). These differences in epidemiology of subtypes of SCAR may be due to either the genetic background of diverse populations or the prevalence of drugs used in clinical practice. 

As previous reports, our results also revealed that antibiotics with a frequency of 44.3% were the most public drug–induced SCARs (8, 14 and 15). Furthermore, beta-lactams with a frequency of 34.4%, especially amoxicillin with a frequency of 11.7%, were the main antibiotics leading to SCARs. Anti-epileptic drugs with a frequency of 36%, especially Phenobarbital with a frequency of 21.3%, were the second cause of SCARs which were in agreement with some previous reports (8, 14 and 15). 

**Table 1:** Characteristics of patients with four major types of severe cutaneous adverse drug reaction

**Patient Characteristics**	**SCARs**				
	**SJS (n = 49)**	**TENS (n = 4)**	**DRESS (n = 6)**	**AGEP (n = 2)**	
Age% (mean ± SD)	7 ± 5	5 ± 4	6 ± 3	10 ± 5	
Sex% (female:male)	24: 25	2: 2	1: 5	0: 2	
Family relationship between parents					
First degree	12.2% (6/49)	0	16.6% (1/6)	0	
Second degree	10.2% (5/49)	75% (3/4)	66.6% (4/6)	50% (1/2)	
Non relative	77.5% (38/49)	25% (1/4)	16.6% (1/6)	50% (1/2)	
Comorbidities					
Atopy	12.2% (6/49)	0	16.6% (1/6)	0	
Autoimmune ds	6.1% (3/49)	0	16.6% (1/6)	0	
Rheumatologic ds	0	0	16.6% (1/6)	0	
Family history					
Familial Autoimmune ds	6.1% (3/49)	0	0	0	
Familial Rheumatologic ds	2% (1/49)	0	0	0	
Infections					
Viral	28.5% (14/49)	25% (1/4)	33.3% (2/6)	50% (1/2)	
Bacterial	16.3% (8/49)	50% (2/4)	16.6% (1/6)	0	
Infection Symptoms					
Respiratory	34.6% (17/49)	50% (2/4)	33.3% (2/6)	50% (1/2)	
GI	6.1% (3/49)	0	16.6% (1/6)	0	
GU	0	25%(1/4)	0	0	
ENT	2% (1/49)	0	16.6% (1/6)	0	
Nervous system	2% (1/49)	0	0	0	
Clinical findings					
Fever	32.6% (16/49)	75% (3/4)	66.6% (4/6)	50% (1/2)	
Rhinitis	18.3% (9/49)	25% (1/4)	16.6% (1/6)	50% (1/2)	
Conjunctivitis	32.6% (16/49)	75% (3/4)	50% (3/6)	50% (1/2)	
Bronchospasm	16.3% (8/49)	25% (1/4)	16.6% (1/6)	0	
Diarrhea and abdominal pain	14.2% (7/49)	75% (3/4)	33.3% (2/6)	0	
Dyspnea and wheezing	14.2% (7/49)	25% (1/4)	33.3% (2/6)	0	
Bloody diarrhea	2% (1/49)	25% (1/4)	0	50% (1/2)	
Nephritis	0	25% (1/4)	0	0	
Hepatitis	6.1% (3/49)	50% (2/4)	83.3% (5/6)	50% (1/2)	
Pneumonitis	2% (1/49)	25% (1/4)	16.6% (1/6)	0	
Rash					
Itching	51% (25/49)	25% (2/4)	83.3% (5/6)	100% (2/2)	
Maculopapular	79.5% (39/49)	100% (4/4)	100% (6/6)	100% (2/2)	
Erythematous	71.4% (35/49)	75% (3/4)	83.3% (5/6)	100% (2/2)
Vesicular	28.5% (14/49)	25% (1/4)	16. 6% (1/6)	100% (2/2)
Morbiliform	28.5% (14/49)	25% (1/4)	33.3% (2/6)	50% (1/2)
Urticaria	26.5% (13/49)	0	0	50% (1/2)
Angioedema	6.1% (3/49)	0	0	50% (1/2)
Hematologic and urinalysis abnormalities				
Leukocytosis	28.8% (13/45)	0	66.6% (4/6)	0
Eosinophilia	4.4% (2/45)	0	100% (6/6)	0
Anemia	24.4% (11/45)	25% (1/4)	50% (3/6)	0
Thrombocytopenia	4.4% (2/45)	0	16.6% (1/6)	0
Elevated ESR	43.5% (17/39)	50% (2/4)	60% (3/5)	50% (1/2)
Abnormal LFT	10.2% (4/39)	25( 1/4)	83.3% (5/6)	50% (1/2)
Coagulopathy	2.7% (1/37)	0	0	0
Positive blood culture	10% (2/20)	50% (1/2)	0	0
Active U/A	8.8% (3/34)	50% (2/4)	60% (3/5)	0
Positive U/C	4% (1/21)	0	0	0
Treatments				
Systemic corticosteroids	11.3% (5/44)	25% (1/4)	0	0
IVIG	0	25% (1/4)	0	0
Anti-histamine	18.1% (8/44)	0	0	0
Corticosteroids + IVIG + Antihistamine	22.7% (10/44)	25% (1/4)	66.6% (4/6)	50% (1/2)
Corticosteroids + IVIG	15.9% (7/44)	25% (1/4)	16.6% (1/6)	50% (1/2)
Antihistamine + Corticosteroids	29.5% (13/44)	0	0	0
Antihistamine + IVIG	2.2% (1/44)	0	16.6% (1/6)	0

SCAR: Severe cutaneous adverse drug reaction; SJS: Stevens-Johnson syndrome; TEN: Toxic epidermal necrolysis; DRESS: Drug reaction with eosinophilia and systemic symptoms; AGEP: Acute generalized exanthematous pustulosis; ESR: Erythrocyte sedimentation rate; LFT: Liver function test.

**Table 2:** Frequency of drugs caused SJS, TEN, DRESS and AGEP in southwestern Iranian patients with SCARs (abbreviations are shown in the footnote of Table 1).

**Drugs**	**SCARs (n = 61)**	**SJS (n = 49)**	**TENS (n = 4)**	**DRESS (n = 6)**	**AGEP (n = 2)**
Beta-lactams	34.4% (21)	42.8% (21)	0	0	0
Amoxicillin	11.4% (7)	14.2% (7)	0	0	0
Penicillin	6.5% (4)	8.1% (4)	0	0	0
Cefixime	4.9% (3)	6.1% (3)	0	0	0
Ceftriaxon	4.9% (3)	6.1% (3)	0	0	0
Cephalexin	3.2% (2)	4% (2)	0	0	0
Co-amoxiclave	1.6% (1)	2% (1)	0	0	0
Cefixime *vs*. Ceftriaxon	1.6% (1)	2% (1)	0	0	0
Non-beta lactams	9.8% (6)	10.2% (5)	25 (1)	0	0
Azithromycin	3.2% (2)	4% (2)	0	0	0
Ciprofloxacin	1.6% (1)	2% (1)	0	0	0
Cotrimoxazole	1.6% (1)	2% (1)	0	0	0
Erythromycin	1.6% (1)	2% (1)	0	0	0
Vincristine *vs. *Actinomycin	1.6% (1)	0	25 (1)	0	0
Anti-epileptics	36% (22)	26.5% (13)	50% (2)	100% (6)	50% (1)
Phenobarbital	21.3% (13)	18.3% (9)	0	50% (3)	0
Phenytoin	4.9% (3)	40.8% (2)	0	16.6% (1)	0
Carbamazepin	3.2% (2)	2% (1)	25% (1)	0	50% (1)
Carbamazepin *vs. *Sodium valproate	1.6% (1)	0	0	16.6% (1)	0
Lamotrigine *vs*. Topiramate	1.6% (1)	2% (1)	0	0	0
Phenobarbital *vs. *Sodium valproate	1.6% (1)	0	0	16.6% (1)	0
Phenytoin *vs*. Sodium valproate	1.6% (1)	0	25% (1)	0	0
Other drugs	11.4% (7)	12.2% (6)	25% (1)	0	50% (1)
Anesthesia	1.6% (1)	2% (1)	0	0	0
Clotrimazole	1.6% (1)	2% (1)	0	0	0
Imipramine	1.6% (1)	2% (1)	0	0	0
Hydroxychlorquine	1.6% (1)	2% (1)	0	0	0
Zidovudine	1.6% (1)	2% (1)	0	0	50% (1)
Clotrimazole + Rifampin	1.6% (1)	0	25% (1)	0	0
Herbal medication	1.6% (1)	2% (1)	0	0	0

**Table 3:** HLA-A and –B allele frequencies in patients with drug hypersensitivity compared to healthy controls

**HLA-A**	**Patients**		**Controls**		***P*** **-value**	**OR**	**CI95%**
	**2n = 80**	**F%**	**2n = 80**	**F%**			
01:01	9	11.25	7	8.75	NS		
01:03	—	—	2	2.5	NS		
02:01	16	20	6	7.5	0.037	3.08	1.1387-8.3491
02:05	—	—	2	2.5	NS		
02:06	—	—	1	1.25	NS		
02:08	—	—	1	1.25	NS		
02:11	—	—	2	2.5	NS		
02:12	—	—	1	1.25	NS		
02:81	2	2.5	—	—	NS		
03:01	4	5	3	3.75	NS		
03:02	2	2.5	6	7.5	NS		
11:01	1	1.25	16	20	0.00013	0.05	0.0065-0.3921
21:02	—	—	1	1.25	NS		
23:01	1	1.25	2	2.5	NS		
24:02	15	18.75	7	8.75	NS		
24:03	—	—	1	1.25	NS		
24:13	—	—	1	1.25	NS		
26:01	5	6.25	1	1.25	NS		
26:09	—	—	1	1.25	NS		
29:01	2	2.5	1	1.25	NS		
29:02	—	—	1	1.25	NS		
30:01	2	2.5	2	2.5	NS		
30:04	—	—	1	1.25	NS		
31:01	5	6.25	—	—	NS		
32:01	5	6.25	2	2.5	NS		
32:04	2	2.5	2	2.5	NS		
33:01	2	2.5	—	—	NS		
33:03	—	—	5	6.25	NS		
36:01	1	1.25	—	—	NS		
36:02	1	1.25	—	—	NS		
43:01	—	—	1	1.25	NS		
68:01	4	5	3	3.75	NS		
68:02	—	—	1	1.25	NS		
68:03	1	1.25	—	—	NS		
**HLA-B**							
07:02	1	1.25	1	1.25	NS		

**Table 4:** HLA-A and –B allele frequencies in patients with hypersensitivity to different drugs compared to healthy controls

Controls			**Hypersensitivity to:**										
			**Anticonvulsant**				**Beta-lactam**				**Non-beta lactam**		
**HLA-A**	**2n = 80**	**F%**	**2n = 34**		**F%**	***P*** **-value**	**2n = 24**		**F%**	***P*** **-value**	**2n = 20**	**F%**	***P*** **-value**
01:01	7	8.75	5		14.71		3		12.50		2	10.00	
01:03	2	2.5	—		—		—		—		—	—	
02:01	6	7.5	3		8.82		7		29.17	0.010	3	15.00	
02:05	2	2.5	—		—		—		—		—	—	
02:06	1	1.25	—		—		—		—		—	—	
02:08	1	1.25	—		—		—		—		—	—	
02:11	2	2.5	—		—		—		—		—	—	
02:12	1	1.25	—		—		—		—		—	—	
02:81	—	—	1		2.94		—		—		—	—	
03:01	3	3.75	1		2.94		—		—		2	10.00	
03:02	6	7.5	1		2.94		1		4.17		—	—	
11:01	16	20	—		—	0.003	—		—	0.020	1	5.00	
21:02	1	1.25	—		—		—		—		—	—	
23:01	2	2.5	—		—		—		—		1	5.00	
24:02	7	8.75	5		14.71		7		29.17	0.017	3	15.00	
24:03	1	1.25	—		—		—		—		—	—	
24:13	1	1.25	—		—		—		—		—	—	
26:01	1	1.25	1		2.94		1		4.17		3	15.00	0.024
26:09	1	1.25	—		—		—		—		—	—	
29:01	1	1.25	1		2.94		—		—		1	5.00	
29:02	1	1.25	—		—		—		—		—	—	
30:01	2	2.5	1		2.94		1		4.17		1	5.00	
30:04	1	1.25	—		—		—		—		—	—	
31:01	—	—	4		11.76	0.007	1		4.17		1	5.00	
32:01	2	2.5	3		8.82		1		4.17		2	10.00	
32:04	2	2.5	—		—		2		8.33		—	—	
33:01	—	—	2		5.88		—		—		—	—	
33:03	5	6.25	—		—		—		—		—	—	
36:01	—	—	2		5.88		—		—		—	—	
36:02	—	—	1		2.94		—		—		—	—	
43:01	1	1.25	—		—		—		—		—	—	
68:01	3	3.75	2		5.88		—		—		—	—	
68:02	1	1.25	—		—		—		—		—	—	
68:03	—	—	1		2.94		—		—		—	—	
07:02	1	1.25	—		—		—		—		1	5.00	
07:05	—	—	—		—		—		—		1	5.00	
07:11	1	1.25	—		—		—		—		—	—	
08:01	2	2.5	4		11.76		—		—		1	5.00	
13:01	1	1.25	—		—		—		—		—	—	
14:02	2	2.5	2		5.88		1		4.17		1	5.00	
15:01	1	1.25	—		—		—		—		—	—	
15:02	—	—	2		5.88		—		—		—	—	
15:03	2	2.5	—		—		—		—		1	5.00	
15:15	—	—	1		2.94		1		4.17		1	5.00	
18:01	6	7.5	1		2.94		2		8.33		2	10.00	
27:05	1	1.25	—		—		—		—		—	—	
27:07	1	1.25	—		—		—		—		—	—	
35:01	9	11.25	3		8.82		4		16.67		2	10.00	
35:02	5	6.25	1		2.94		1		4.17		—	—	
35:03	5	6.25	—		—		—		—		—	—	
35:10	1	1.25	—		—		—		—		—	—	
35:34	1	1.25	—		—		—		—		—	—	
38:01	1	1.25	1		2.94		—		—		3	15.00	0.024
39:06	—	—	1		2.94		—		—		—	—	
40:06	3	3.75	—		—		—		—		—	—	
41:01	—	—	1		2.94		3		12.50	0.011	1	5.00	
44:02	3	3.75	2		5.88		2		8.33		1	5.00	
44:26	1	1.25	—		—		—		—		—	—	
45:01	1	1.25	1		2.94		—		—		—	—	
49:01	2	2.5	—		—		—		—		—	—	
50:01	5	6.25	—		—		1		4.17		1	5.00	
50:02	—	—	1		2.94		—		—		—	—	
51:01	5	6.25	11		32.35	0.0006	6		25.00	0.017	3	15.00	
51:06	1	1.25	—		—		—		—		—	—	
51:08	—	—	1		2.94		—		—		—	—	
52:01	7	8.75	—		—		—		—		—	—	
55:01	2	2.5	1		2.94		2		8.33		—	—	
55:02	1	1.25	—		—		—		—		—	—	
56:01	1	1.25	—	—			1	4.17			—	—	
57:01	4	5	—	—			—	—			—	—	
57:11	1	1.25	—	—			—	—			—	—	
58:01	2	2.5	—	—			—	—			—	—	
73:01	—	—	—	—			—	—			1	5.00	
78:06	1	1.25	—	—			—	—			—	—	

**Table 5:** HLA-A and –B allele frequencies in patients with Stevens–Johnson syndrome (SJS) compared to healthy controls

**HLA-A**		**SJS**		**Controls**			***P*** **-value**	**OR**	**CI95%**
		**2n = 64**	**F%**	**2n = 80**		**F%**			
01:01		8	12.50	7		8.75	NS		
01:03		—	—	2		2.5	NS		
02:01		13	20.31	6		7.5	0.024	3.1438	1.1212-8.8150
02:05		—	—	2		2.5	NS		
02:06		—	—	1		1.25	NS		
02:08		—	—	1		1.25	NS		
02:11		—	—	2		2.5	NS		
02:12		—	—	1		1.25	NS		
02:81		1	1.56	—		—	NS		
03:01		2	3.13	3		3.75	NSS		
03:02		2	3.13	6		7.5	NS		
11:01		1	1.56	16		20	0.0007	0.0635	0.0082-0.4932
21:02		—	—	1		1.25	NS		
23:01		1	1.56	2		2.5	NS		
24:02		15	23.44	7		8.75	0.015	3.1924	1.2133-8.3999
24:03		—	—	1		1.25	NS		
24:13		—	—	1		1.25	NS		
26:01		3	4.69	1		1.25	NS		
26:09		—	—	1		1.25	NS		
29:01		2	3.13	1		1.25	NS		
29:02		—	—	1		1.25	NS		
30:01		2	3.13	2		2.5	NS		
30:04		—	—	1		1.25	NS		
31:01		3	4.69	—		—	NS		
32:01		3	4.69	2		2.5	NS		
32:04		2	3.13	2		2.5	NS		
33:01		2	3.13	—		—	NS		
33:03		—	—	5		6.25	NS		
36:01		1	1.56	—		—	NS		
36:02		1	1.56	—		—	NS		
43:01		—	—	1		1.25	NS		
68:01		2	3.13	3		3.75	NS		
68:02		—	—	1		1.25	NS		
**HLA-B**									
07:02		—	—	1		1.25	NS		
07:05		1	1.5625	—		—	NS		
07:11	—		—		1	1.25	NS			
08:01	5		7.8125		2	2.5	NS			
13:01	—		—		1	1.25	NS			
14:02	4		6.25		2	2.5	NS			
15:01	—		—		1	1.25	NS			
15:02	2		3.125		—	—	NS			
15:03	1		1.5625		2	2.5	NS			
15:15	2		3.125		—	—	NS			
18:01	4		6.25		6	7.5	NS			
27:05	—		—		1	1.25	NS			
27:07	—		—		1	1.25	NS			
35:01	7		10.9375		9	11.25	NS			
35:02	2		3.125		5	6.25	NS			
35:03	—		—		5	6.25	NS			
35:10	—		—		1	1.25	NS			
35:34	—		—		1	1.25	NS			
38:01	2		3.125		1	1.25	NS			
40:06	—		—		3	3.75	NA			
41:01	5		7.8125		—	—	0.016			
44:02	4		6.25		3	3.75	NS			
44:26	—		—		1	1.25	NS			
45:01	1		1.5625		1	1.25	NS			
49:01	—		—		2	2.5	NS			
50:01	3		4.6875		5	6.25	NS			
51:01	16		25		5	6.25	0.0015	5.0000	1.7192-14.5413	
51:06	—		—		1	1.25	NS			
52:01	—		—		7	8.75	0.017			
55:01	2		3.125		2	2.5	NS			
55:02	—		—		1	1.25	NS			
56:01	1		1.5625		1	1.25	NS			
57:01	—		—		4	5	NS			
57:11	—		—		1	1.25	NS			
58:01	—		—		2	2.5	NS			
73:01	2		3.125		—	—	NS			
78:06	—		—		1	1.25	NS			

*SJS/TEN*

In our study, the mean age of the patients with SJS and TEN was 7 and 5 years, respectively, and girl to boy ratio was equal in both diseases. Our data are in consistent with previous studies which reported the peak age of presentation of these diseases between 1-10 years with no gender preference (16). The most common comorbidity in patients with SJS was atopy (12.2%) which may indicate the existence of a common immunopathology (17). 

Furthermore, it was manifested that about 28.3% of the patients with SJS/TEN had viral infection and 18-8% had bacterial infection which were both mainly presented by respiratory symptoms (35.8%). Previous studies also reported the presentation of SJS/TEN in patients following viral infections (18, 19). Moreover, infection was found to be a risk factor for severe symptoms and early onset of SJS/TEN (20). 

Two most common clinical presentations of our patients with SJS and TEN were fever and conjunctivitis. Fever was reported as an initial symptom even before skin manifestations (21), while ocular presentation *e.g.* conjunctivitis, was a late sign in the course of these diseases (22). The most common skin lesion in SJS/TEN was pruritic erythematous maculopapular rash. Skin eruption in SJS was previously described as small blisters arising on purple macules. Skin presentation in TEN was also defined as large blisters having positive Nikolski sign (5). 

The most common laboratory finding in our patients with SJS and TEN was elevated acute phase reactants led to inflammatory process, which contribute in pathophysiology of both diseases (23). 

Regarding to our results, the most common drug–induced SJS were beta-lactams especially amoxicillin. On the other hand, the most common drug causing TEN in our patients was anti-epileptics especially carbamazepine. Sulfonamide antibiotics and anti-epileptic drugs were also previously reported to be associated with higher risk of SJS/TEN; however, some of the surveys gave priority to anti-epileptics in both SJS and TEN (5, 24). These controversies in the frequency of drug–induced SJS/TEN can be due to differences in the frequency of drugs used clinically in each country which is mainly due to the prevalence of various diseases in that area. 

*DRESS*

DRESS was diagnosed in six patients that in two of them it was following viral infection mostly presented by respiratory symptoms. Some other studies also reported the onset of DRESS in patients following viral infections such as EBV, HIV, or herpes virus (25, 26). 

Most common clinical presentations of DRESS among our patients were hepatitis, fever and conjunctivitis. Fever, lymphadenopathy and hematologic abnormalities were previously reported as three major manifestations of DRESS while visceral organ involvement such as hepatitis or ocular manifestations such as conjunctivitis had been reported with lower frequencies (5). The most common skin lesions in this disease were pruritic erythematous maculopapular skin rash. Previous studies, however, mainly mentioned exanthema as the most common skin eruption in DRESS (5, 27). Analysis of our patients with DRESS showed eosinophilia, increases liver function tests (AST, ALT), leukocytosis, elevated acute phase reactants and anemia. Although the RegiSCAR considered the presence of atypical lymphocytes as one of the evidence in favor of DRESS (28) and Cornell *et al.* was also mentioned to the presence of 9% atypical lymphocytes in the peripheral blood of a 29-year-old Asian female diagnosed as DRESS (29), we did not find atypical lymphocytes in blood smears of any of our six patients with DRESS.

All of our patients showed DRESS symptoms following administration of anti-epileptic drugs. Three of them were under treatment with phenobarbital, one with phenytoin, one with carbamazepine and sodium valproate, and the other one with phenobarbital and sodium valproate. Our results are in consistent with previous reports which also considered anti-epileptics as the most common drugs involved in DRESS; however, minocycline, allopurinol, and sulfonamides were also mentioned as the etiology of DRESS (5). 

*AGEP*

We had two patients diagnosed with AGEP. Both of them were male with mean age of 10 years. Parents of one the patients were first-degree relatives while the parents of another patient were unrelated. Furthermore, the major clinical findings of these patients were fever, conjunctivitis, rhinitis, hepatitis, and bloody diarrhea. Skin lesions in these patients were pruritic erythematous vesicular and maculopapular lesions. Previous studies, however, emphasized on the presence of fever and pustular skin eruptions in patients with AGEP (30). Although leukocytosis especially neutrophilia was considered the main abnormal laboratory data in AGEP, our results demonstrated that rise in acute phase reactants and abnormal liver function tests were the most common ones (31). 

Although antibiotics such as aminopenicillins and diltiazem were previously reported to be the most common drugs leading to AGEP, anti-epileptic drugs such as carbamazepine and zidovudine were the most common drugs caused AGEP in our patients (5). 

*SCAR Treatment*

In addition to cessation, the main SCAR treatments in our center were corticosteroids, antihistamines, and IVIG. However, some previous studies insisted on supportive therapy as the first therapeutic line for all kinds of SCARs (32). Some researchers suggested using high potency topical or systemic steroids as the pivotal treatment of SCARs (25, 33). Although no benefit has been reported in using of IVIG for the treatment of any types of DHRs, especially SJS/TEN (34, 35), most of our patients showed promising responses to IVIG in combination with corticosteroids and/or antihistamines.

*HLA Association of DHRs*

As it was mentioned previously, adverse cutaneous drug reactions are side effects of medicines which are thought to be idiosyncratic and unpredictable (36). One of the major causes is individual genetic variations in drug metabolism (37). Hence, recently, some of the studies on adverse drug reactions including SCARs focused on relation of these drug side effects presentations with certain human leukocyte antigen (HLA) alleles especially HLA-A and HLA-B (38). 

Our results demonstrated that the frequency of HLA-A*02:01 (20% *vs* 7.5%, *P* = 0.037, OR = 3.08, CI95%: 1.1387-8.3491) and HLA-B*51:01 (23.75% *vs.* 6.25%, *P* = 0.003, OR = 4.64, CI95%: 1.6489-13.2382) alleles were significantly higher in patients than controls. These results also indicated that the patients who were positive for HLA-A*02:01 allele were 3.08 times, and those carried HLA-B*51:01 allele were 4.67 times more susceptible to SCARs than the individuals who did not have these alleles. Whereas, HLA-A*11:01 (*P *= 0.00013) and HLA-B*52:01 (*P *= 0.013) were significantly higher in controls rather than patients. 

Previous studies, otherwise, reported different alleles contributing in SCARs such as HLA-B*15:02 and HLA-B*57:01 which were shown to be related to Carbamazepine–induced SJS/TEN, abacavir–induced hypersensitivity and flucloxacillin–induced liver injury (32). Carbamazepine–induced SJS/TEN in HLA-B*15:02 bearing patients and allopurinol–induced SCAR in HLA-B*58:01 carriers were also reported by Chung *et al.* (39). 

Our results also showed a positive association between HLA-A*11:01 allele and anticonvulsant–induced SCAR (*P* = 0.003) while, HLA-A*31:01 allele was negatively associated to anticonvulsant–induced SCAR (*P* = 0.007). Contrary to our results, Kim *et al.* found an association between HLA-A*31:01 allele and lamotrigine–induced SCAR in Korean patients (40), and Kay *et al.* also found a positive link between this allele and carbamazepine–induced rash in German patients (41). Nevertheless, other studies reported association between HLA-B*40:02 or HLA-B*15:02 alleles with SCARs induced by anticonvulsants especially carbamazepine (42-44). 

Our results also revealed that HLA-A*02:01 (*P* = 0.01), HLA-A*24:02 (*P *= 0.017), HLA-B*41:01 (*P* = 0.011), and HLA-B*51:01 (*P *= 0.017) were significantly higher in patients with hypersensitivity to beta-lactams than controls; whereas HLA-A*11:01 showed a significant lower frequency in patients compared to controls (*P* = 0.02). Same study on non-beta lactams demonstrated that HLA-A*26:01 and HLA-B*38:01 were significantly higher in patients compared to controls (*P* = 0.024 for each allele). In contrast to our results, an association between HLA-B*15:02 and cotrimoxazole–induced SCAR was reported by Kongpan *et al.* (45), and levofloxacin–induced SJS/TEN was also reported in a patient with HLA-DRB1*03:01 and DQB1*02:01 alleles (46). 

Our data revealed positive associations between HLA-A*02:01 (*P* = 0.024), HLA-A*24:02 (*P *= 0.015), and HLA-B*51:01 (*P *= 0.001) alleles with SJS which increased the risk of the disease development around 3 to 5 times in our patients. While, HLA-A*11:01 showed a protective role against SJS development (OR 0.06). HLA-A*31:01 and HLA-B*15:02 were previously reported to be related to carbamazepine–induced SJS (47). HLA-A*33:03 and HLA-C*03:02 alleles were previously reported to have association with allopurinol–induced SJS/TEN in Asians (48). 

These conflicts between our results and previous reports show the genetic variability in different populations. Confirming of the association of certain HLA allele (s) with specific drugs remains to be explained by expanding genetic studies on more patients from different populations (49, 50). 

## Conclusion

Based on our results, beta-lactam antibiotics and anti-epileptic drugs are the most common causes of severe adverse drug reactions in southwestern Iranian pediatric patients. Moreover, HLA-A*02:01 and A*51:01 alleles can be considered as predisposing genetic factor for SCAR.
